# Interfacial Coupling and Modulation of van der Waals Heterostructures for Nanodevices

**DOI:** 10.3390/nano12193418

**Published:** 2022-09-29

**Authors:** Kun Zhao, Dawei He, Shaohua Fu, Zhiying Bai, Qing Miao, Mohan Huang, Yongsheng Wang, Xiaoxian Zhang

**Affiliations:** Key Laboratory of Luminescence and Optical Information, Ministry of Education, Institute of Optoelectronic Technology, Beijing Jiaotong University, Beijing 100044, China

**Keywords:** van der Waals heterostructure, interfacial interaction, modulation, devices

## Abstract

In recent years, van der Waals heterostructures (vdWHs) of two-dimensional (2D) materials have attracted extensive research interest. By stacking various 2D materials together to form vdWHs, it is interesting to see that new and fascinating properties are formed beyond single 2D materials; thus, 2D heterostructures-based nanodevices, especially for potential optoelectronic applications, were successfully constructed in the past few decades. With the dramatically increased demand for well-controlled heterostructures for nanodevices with desired performance in recent years, various interfacial modulation methods have been carried out to regulate the interfacial coupling of such heterostructures. Here, the research progress in the study of interfacial coupling of vdWHs (investigated by Photoluminescence, Raman, and Pump–probe spectroscopies as well as other techniques), the modulation of interfacial coupling by applying various external fields (including electrical, optical, mechanical fields), as well as the related applications for future electrics and optoelectronics, have been briefly reviewed. By summarizing the recent progress, discussing the recent advances, and looking forward to future trends and existing challenges, this review is aimed at providing an overall picture of the importance of interfacial modulation in vdWHs for possible strategies to optimize the device’s performance.

## 1. Introduction

Along with the rise of graphene, alternative layered two-dimensional (2D) materials, which consist of strong in-plane covalent bonds and weak intra-plane van der Waals bonds, have attracted tremendous attention all around the world. The easy exfoliation into stable atomically thin materials, together with the dimensionality effect, induced fascinating properties, enabling the construction of various devices with smaller sizes and fewer defects [1]. Especially, 2D materials, such as transition metal dichalcogenides (TMDs), transition metal carbides or carbon-nitrides (Mxenes), and boron nitride (BN) [2,3,4], are extremely thin, transparent, and flexible, allowing for transparent devices to be made in flexible circuit materials [5,6,7]. TMDs have excellent properties, such as large surface area, great mechanical properties, high mobility, large exciton binding energy and long exciton lifetime, etc., thus providing unprecedented access to the exploration of new physics in such quantum systems, and meanwhile promote the applications of electronic and optoelectronic devices [8,9,10]. Currently, various devices with high performance have been developed based on 2D materials, such as field effect transistors (FETs) for logic circuits, radio-frequency devices, chemical sensing, optoelectronic devices, memristors, etc. [10,11,12,13]. The research progress of 2D materials, including the synthesis of 2D materials, the exploration of new physics, and the applications of various devices, has been comprehensively reviewed by many papers [10,14,15,16,17,18] and inspired a lot of researchers in related fields.

With the development of 2D materials, researchers have focused on the study of the van der Waals (vdW) heterostructure due to the increasing demand for excellent junctions in the semiconductor industry. Different from traditional semiconductor heterojunctions, high-quality vdW heterostructures (vdWHs) can be fabricated by simply stacking two layers of 2D materials together, similar to Lego [16,19,20,21,22]. Note that, obviously, more layers of 2D materials can be stacked together to build more complicated vdWHs if necessary, but currently, most of the research work still focuses on the vdWHs consisting of two kinds of materials. Integrating different 2D materials to form various vdWHs can effectively broaden the variety of physical properties and create novel device structures without considering the lattice matching in highly different material systems. Furthermore, inspired by such 2D/2D TMD vdWHs, organic semiconductors with similar conjugated structures have been utilized to fabricate organic–inorganic vdWHs, which also holds great potential for building multifunctional flexible nanodevices with even higher performance for future electronics and optoelectronics. At present, various devices based on such 2D-structured vdWHs (including both inorganic 2D/2D vdWHs and organic–inorganic vdWHs), such as new types of solar cells and photodetectors [23,24,25], photoelectric memories [26,27,28] and other two-dimensional devices [29,30,31,32], have been reported.

However, the realization of such electronic and optoelectronic devices is dominated by the fundamental understanding of the interface. In order to achieve the predictable utilization of vdWHs in such devices, the understanding and control of the key parameters in the device metrics must be solved. Therefore, it is necessary to characterize the actual electronic structure of the heterostructure interfaces, such as interfacial dipole interaction, local charge transfer, and the resulting kinetic processes, such as the carrier relaxation paths and the decay times, and explore the methods that can effectively modulate these microscopic processes to optimize the physical properties and thus improve the device performance.

**Figure 1:**
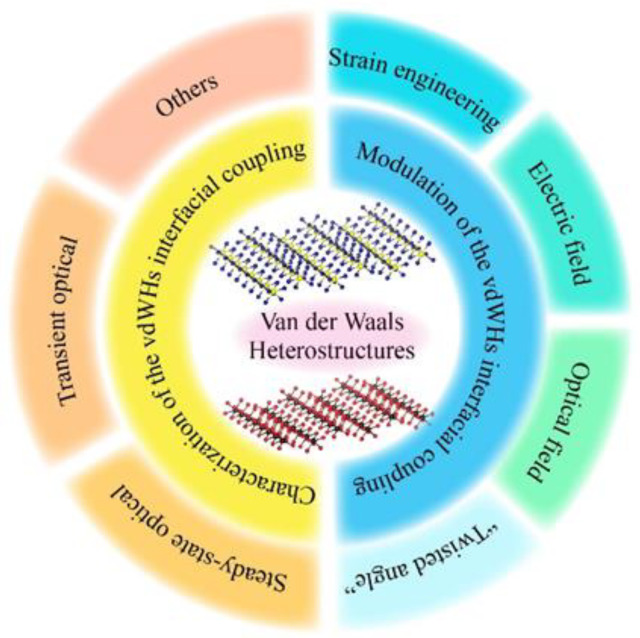
Schematic illustration of the main contents in this review.

Herein, we review the latest research progress in the interfacial coupling of vdWHs, including inorganic 2D heterostructures and organic–inorganic heterostructures. First, research progress in the various methods to fully characterize the interfacial coupling of vdWHs was summarized, including (1) stable-state optical methods such as optical absorption, photoluminescence (PL), and Raman spectroscopies, (2) transient optical methods, such as pump–probe spectroscopy, transient absorption spectroscopy, and transient electronic sum frequency generation spectroscopy, etc., and (3) other techniques such as Kelvin probe force microscopy (KPFM), scanning tunneling microscopy (STM), piezoresponse force microscopy (PFM) et al. Moreover, we also review the recent modulation strategies of the interfacial coupling of vdWHs. The modulation part is divided into four parts: (1) strain engineering modulation, (2) electric field modulation, (3) optical field modulation, and (4) “twisted angle” modulation. In order to make this review paper easier to understand, we have drawn the main structure of this review in the schematic illustration shown in Figure 1. Generally, this paper only briefly summarizes the research progress related to vdWHs interfacial coupling, hoping to provide some basic information for researchers or students who are new to this field.

## 2. Various Characterization Methods to Investigate the Interfacial Coupling of vdWHs

**Figure 2:**
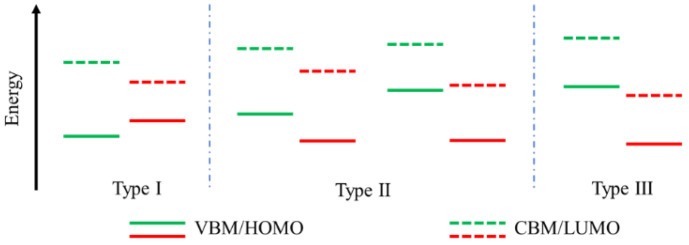
Schematic of type I, type II, and type III arrangements of heterostructures.

Heterostructures are widely utilized as a basic building block in advanced semiconductor devices, such as field effect transistors (FET), light-emitting diodes, and so on. Generally, there are three types of heterostructures, i.e., type І, type ІІ, and type ІІІ (Figure 2). Type I arrangements can be used for energy transfer and are widely applied in organic light-emitting diodes (OLEDs) [33]. Whereas the type II arrangements enable efficient electron/hole separation at interfaces of optoelectronic devices, such as photodetectors [34], photodiodes [35], and photovoltaics [36]. Compared to the other two heterostructures, however, the type III arrangements are less common and rarely utilized in the semiconductor industry [37]. Since this review paper targets the potential applications of vdWHs for further electronics and optoelectronics, we will mainly focus on the type II arrangements here. Some of the identified types of vdWHs in the review are listed in Table 1. In order to apply vdWHs to practical electric and optoelectronic applications, it is essential to have a clear understanding of interfacial physics, such as interlayer coupling, charge transfer, exciton formation, and dynamics. By fully characterizing the electronic structure, optical response, and optoelectronic properties, including interfacial energy level alignment without and under illumination, charge transfer, light absorption, and emission, etc., we could obtain key information for device design and optimization in the future. Currently, various techniques, including optical absorption spectroscopy, Raman spectroscopy, photoluminescence spectroscopy, transient optical technology, atomic force microscopy (AFM), multi-function scanning probe microscopy, and transmission electron microscopy (TEM), etc., are widely utilized as powerful tools for fully characterize the interfacial coupling of vdWHs. Here, we will start with the general characterization techniques commonly used to characterize the interfacial coupling of vdWHs. i.e., both steady-state and transient optical characterization methods, which are discussed first in Section 2.1 and Section 2.2. In addition, we will summarize some research progress of some other techniques, including SPM, TEM, etc., in Section 2.3.

### 2.1. Steady-State Optical Characterization

In order to achieve a fundamental understanding of the interfacial coupling of vdWHs, it is essential to know the interfacial electronic structures, such as band alignment, new electronic states after interfacial coupling, defects, and so on. Moreover, the formation of high-quality atomic layers provides a perfect platform to explore intriguing physics that is not easy to observe in other systems. For instance, the stable formation of biexciton, dark excitions, and trions at room temperature have been reported in some vdWHs [64]. In the past few years, optical absorption, PL, Raman, etc., optically related characterization techniques have proven to be very powerful tools to provide abundant information about the interfacial coupling of vdWHs, such as electronic structure, charge transfer, local strain, electron–phonon interaction, and multi-body effect, etc., at the interface [65,66]. Since these optical techniques can be easily operated under an ambient atmosphere by using advanced focusing systems together with a great laser source; however, optical spectra with high spectral resolution and high spatial resolution can be detected on vdWHs. In addition, equipped with a temperature control system, time-dependent spectra can also be obtained to provide more information about the interfacial coupling of vdWHs, such as the coupling between charge and spin, electron–phonon interactions, and order and disorder states in vdWHs. Note that sometimes ultrafast laser can also be utilized as an excitation source in some of these measurements, but CW laser or other CW light sources are utilized commonly for these measurements, so we will focus on the cases of using steady-state light sources here.

**Figure 3:**
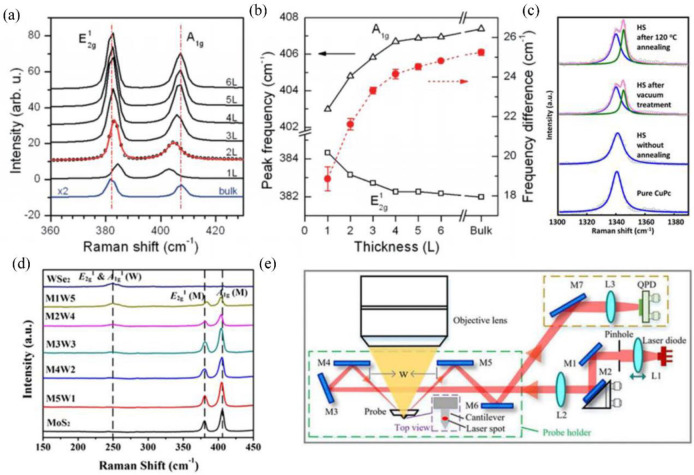
(**a**) Raman spectra of MoS_2_ [71]. (**b**) Variation of frequencies of $E_{2g}^{1}$ and A_1g_ Raman modes with layer thickness. Reprinted with the permission of Ref. [71]. Copyright 2010, American Chemical Society. (**c**) Raman spectra of the CuPc film and MoSe_2_/CuPc HS. Reprinted with permission from Ref. [38] Copyright 2021, American Chemical Society. (**d**) Raman spectra of MoS_2_, WSe_2_, and heterostructure. Reprinted with permission from Ref. [41]. Copyright 2020, Elsevier. (**e**) The diagram of OBD light path in the AFM head. Reprinted with the permission of Ref. [73]. Copyright 2022, AIP Publishing.

Raman spectroscopy is an effective non-destructive method used to study the atomic layer number, built-in strain, doping level, and structural defects in TMD materials [67,68,69,70]. It is also commonly used to examine film thickness and structural properties and determine if a heterostructure has been formed. First, Raman spectroscopy is commonly used to confirm the number of layers in the TMD samples. The number of layers of the TMD material is determined by the distance between the positions of the two fingerprint peaks. The corresponding number of layers can be obtained by calculating the distance of the peak positions. Lee et al. characterized single-layer and few-layer MoS_2_ films by AFM and Raman spectroscopy [71]. They found that the $E_{2g}^{1}$ mode frequency decreased, and the $A_{1g}$ mode frequency increased with increasing layer thickness. This is shown in Figure 3a,b. This work illustrates that the frequency difference between the $E_{2g}^{1}$ mode and the A_1g_ mode can be used as a reliable and convenient diagnostic method for the layer thickness of the MoS_2_ samples. In addition, the Raman spectra can reflect the information about interfacial coupling by the appearance of new peaks or the splitting of the original peaks in the spectra. For instance, our previous work found a new Raman mode at ∼1345 cm^−1^ in the MoSe_2_/CuPc heterostructure due to the strong interfacial coupling between the two materials [38], as shown in Figure 3c. Furthermore, it is worth mentioning that Raman characterization could evaluate the coupling strength of heterojunctions effectively and nondestructively. Moreover, the emergence of Raman modes in low wavenumber regions can also be used to characterize interfacial coupling. Lin et al. demonstrated that the Raman enhancement of the LB modes in hBN/WS_2_ vdWHs is caused by component- vdWH electron–phonon coupling (EPC) mediated by interfacial coupling between the hBN and WS_2_ components [39]. This work reports strong cross-dimensional coupling between layer-breathing phonons and electrons in the minority-layer WS_2_ component, which can be well extended to tens to hundreds of layers of vdWH. The strength of this cross-dimensional EPC can be well reproduced in vdWHs by interfacial coupling and modulation of the interlayer bonding polarizability model. This work provides an effective method for the study of interfacial coupling and EPC in vdWHs. In addition, the quality of the WS_2_/MoSe_2_ heterostructure can be examined using Raman spectroscopy [40]. It was found that the $A_{1g}$ mode of WS_2_ was sensitive to the layer thickness and was found to be greatly enhanced, indicating the successful formation of a heterogeneous layer. In addition, Si et al. found that the $E_{2g}^{1}$ mode in the heterogeneous structure is blue-shifted, and the A_1g_ mode is red-shifted compared to the pure MoS_2_ in Figure 3d. The distance between the two modes decreases, which is related to the number of specimen layers and the peeling effect. This suggests that the formation of the MoS_2_/WSe_2_ heterostructure hinders the composite of multilayer MoS_2_ nanosheets under the vdW force [41]. Furthermore, Amsterdam et al. [72] used Raman spectroscopy to study the growth direction of pentacene on different substrates. The ratio of the intensities of the two vibrational modes, B_3g_ (1597 cm^−1^) and A_g_ (1533 cm^−1^), used in the Raman spectrum, confirms that the pentacene films grown along the π-face on the hBN, while on SiO_2_, they are grown along the edges perpendicular to the SiO_2_ substrate. Furthermore, temperature-dependent Raman experiments can provide more information about the interlayer coupling of vdWHs. For instance, Chen et al. used the temperature dependence of PL and Raman spectra together to study the interlayer coupling and energy band structure of MoS_2_/TaS_2_ heterostructures [42]. The Raman results show that the interlayer coupling leads to the softening of the phonon vibrations in MoS_2_/TaS_2_ heterostructures. To sum up, Raman spectroscopy is a very powerful tool that is capable of determining the number of layers in the TMD samples, evaluating the coupling strength, and analyzing the coupling modes of vdWHs.

However, one can see that in most of the discussions above, only one characterization technique, Raman spectroscopy, was used to provide information about interfacial coupling in vdWHs. As an optical technique, the lateral resolution of Raman spectroscopy is restricted by the diffraction limit of light. Compared with Raman spectroscopy, AFM is capable of providing useful information for surface topology, physical property measurement, and manipulation with unprecedented resolution. The combination of Raman spectroscopy and AFM is considered to be a feasible solution not only to achieve both high resolution and efficiency at the same time but to provide more comprehensive information about the physical picture of the materials. The development of tip-enhanced Raman spectroscopy (TERS) is an excellent example. TERS allows the detection of weak Raman signals with nanometer spatial resolution by using a novel metal tip to provide a strong local plasma field, which is unreachable with common Raman detection [74,75]. Very recently, the Wu group has developed a novel thin head AFM that can be integrated with a Raman microscope to provide the fast and high-resolution measurement of 2D materials [73]. As far as we know, however, the reports of the applications of these techniques in the investigation of the interfacial coupling of vdWHs are rare. In the near future, it is expected that more details will be seen about the roles of the defects, absorption of environmental molecules, as well as local strain, etc., in the interfacial coupling of vdWHs by utilizing these new techniques, which will definitely broaden our understanding about the insights into vdW interfacial coupling.

**Figure 4:**
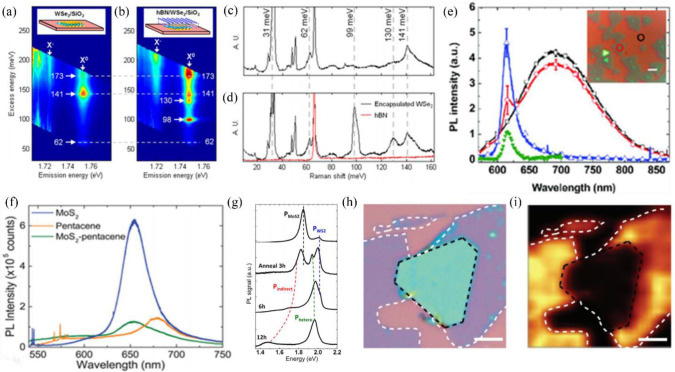
(**a**,**b**) PLE intensity plots of WSe_2_/SiO_2_ and hBN/WSe_2_/SiO_2_ [76]. (**c**) Raman spectrum of WSe_2_/SiO_2_ sample [76]. (**d**) Raman spectra from hBN/WSe_2_/SiO_2_ sample and hBN. Reprinted with permission from Ref. [76] Copyright 2017, American Chemical Society. (**e**) Photoluminescence spectra of PTCDA, WS_2_, and PTCDA/WS_2_ Hs. The inset shows the sample under an optical microscope. Reprinted with permission from Ref. [35] Copyright 2017, American Chemical Society. (**f**) Photoluminescence spectra of MoS_2_, pentacene films, and MoS_2_-pentacene Hs. Reprinted with permission from Ref. [36]. Copyright 2017, American Chemical Society. (**g**) PL of WS_2_/MoS_2_ heterostructures after annealing. Reprinted with permission from Ref. [45]. Copyright 2014, American Chemical Society. (**h**) Optical image of WSe_2_/PbI_2_. [46] (**i**) The photoluminescence mapping image of the same heterostructure shown in (**h**). Reprinted with permission from Ref. [46] Copyright 2019, WILEY-VCH Verlag GmbH &. Co.kGaA.Weinheim.

It is commonly known that both PL and Raman signals can be detected simultaneously, so sometimes, what is observed in the PL measurement may also be Raman peaks; one should be careful about this. However, combining PL and Raman results can provide more information about the interfacial coupling in vdWHs. For example, the Xu group reported the unusual interfacial coupling in hBN/WSe_2_/SiO_2_ heterostructures by utilizing the photoluminescence excitation (PLE) mapping (shown in Figure 4a,b) [76]. They found that the hBN/WSe_2_/SiO_2_ structure has two PLE peaks appearing at excess energies of 130 and 98 meV than that of the WSe_2_/SiO_2_ sample. Two new Raman signals were found by Raman testing in the hBN/WSe_2_/SiO_2_ structures at 130 meV and 98 meV (as shown in Figure 4c,d), matching the corresponding peaks in the PLE intensity plots. Just as we saw, PL is also a common characterization method to reveal the electronic structure and interface states at the interface of the heterostructure. One can gain information by detecting the change of PL intensity or PL peak position in the spectrum. The interfacial charge transfer can be inferred from the PL quenching. For example, Han et al. observed photoluminescence quenching in the WS_2_/ReSe_2_ heterostructure, indicating effective interlayer charge transfer [43]. Similarly, He et al. found no PL quenching in the 2L-In_2_Se_3_/1L-MoSe_2_ heterostructure by comparison, indicating that the interlayer charge transfer was not significant, suggesting that 2L In_2_Se_3_ and 1L MoSe_2_ did not form a type-Ⅱ energy band arrangement [44]. A heterostructure consisting of monolayer WS_2_ and PTCDA films was prepared by Liu et al. [35]. The PL intensities of WS_2_ and PTCDA in the heterostructure in Figure 4e are reduced by 12 ± 5% and 80 ± 10%, respectively, indicating that excitons generated by PTCDA and WS_2_ are free at the heterogeneous interface. The blue line in Figure 4f indicates that the MoS_2_ monolayer is mainly emitted by an exciton at 654 nm, and the broad peaks at 585 nm and 680 nm appear in the pentacene films. In the MoS_2_/pentacene heterostructure, the PL from the MoS_2_ exciton is burst by 83% [36]. Therefore, the weakening of the PL peak intensity at the interface is a measure of whether an effective charge transfer is occurring at the interface of the heterojunction. In addition, the shift of the peak positions and the appearance of new peaks in the PL spectrum are also very useful for evaluating the interfacial coupling of vdWHs. In Figure 4g, Tonay et al. found that the intensity of P_MoS2_ in the WS_2_/MoS_2_ heterostructure is 5–10 times higher than that of P_WS2_, with a new PL peak (P_hetero_) at 1.94 eV, and its integral intensity increases with increasing annealing time. P_MoS2_ and P_WS2_ gradually decreased after annealing. Another weak emission peak, P_indirect_, appears at 1.75 eV at 3 h of annealing, and the peak position is red-shifted with the increased time. The results indicate that the enhanced interlayer coupling also has an effect on the exciton luminescence of the heterostructure, and the degree of coupling can be externally adjusted by vacuum annealing [45]. Recently, PL measurements using a polarized laser as an excitation source have been applied to study the interfacial coupling in the vdWHs. For instance, circularly polarized light with left and right spins has been used to study the interaction between electrons and phonons and to detect spin currents. Circularly polarized PL measurement has been carried out to detect valley-specific interlayer excitons, which has been proved to be a useful tool for exploring spin/valley tronic applications based on vdWHs [77,78]. In addition, based on calculating photoluminescence quantum yields, one can evaluate the quantum efficiency of vdWHs, and time-resolved photoluminescence has found that the lifetime of valley excitons in monolayer TMD in different samples ranges from a few picoseconds to several hundred picoseconds [79,80,81,82]. Except for the PL spectrum, PL mapping can visualize the observation of interlayer coupling of vdWHs. One can easily see the PL quenching effect from different regions of WS_2_/PbI_2_ heterostructures by comparing the contrasts of optical images and PL maps (Figure 4h,i). The WSe_2_/PbI_2_ region is significantly darker compared to the brighter region of monolayer WSe_2_ [46]. Except for the measurement at room temperature, temperature-dependent PL measurement provides more information about the assignments of various excitons and the new electronic states generated by interfacial coupling. For example, Meng et al. reported that after annealing of GaS/MoS_2_ vdWHs at 300 °C under vacuum conditions, the strong coupling interaction between the two layers leads to the changes in the positions and intensities of the emission peaks [47]. This strong coupling interaction is further confirmed by low-temperature PL measurements, i.e., the peak intensities in the spectrum of GaS/MoS_2_ vdWHs decrease with increasing temperature, which is due to the thermal ionization of bound excitons to free excitons at higher temperatures.

As can be seen from the above, PL measurement is a nondestructive and powerful test method not only for characterizing defects and impurities in vdWHs quickly and easily but can explore the exciton behaviors, such as interlayer exciton, hybrid exciton, multi-body effects, etc., in vdWHs. Similar to Raman and other optical microscopic techniques, the lateral resolution of the PL measurement is also only a few hundred of nanometers due to the diffraction limit of light. In order to obtain a clearer picture of the origin of the strong interlayer coupling in vdWHs, working together with other high-resolution techniques such as TEM and AFM will be a smart choice. Introducing light into the high vacuum chamber to achieve TEM-PL/Raman as well as AFM-PL/Raman measurements simultaneously is on the road. The applications of these systems to explore the interfacial coupling in vdWHs will provide more insights into the microscopic working mechanisms, such as analyzing the local electron–phonon interaction and capturing new types of excitons and even phonons.

**Figure 5:**
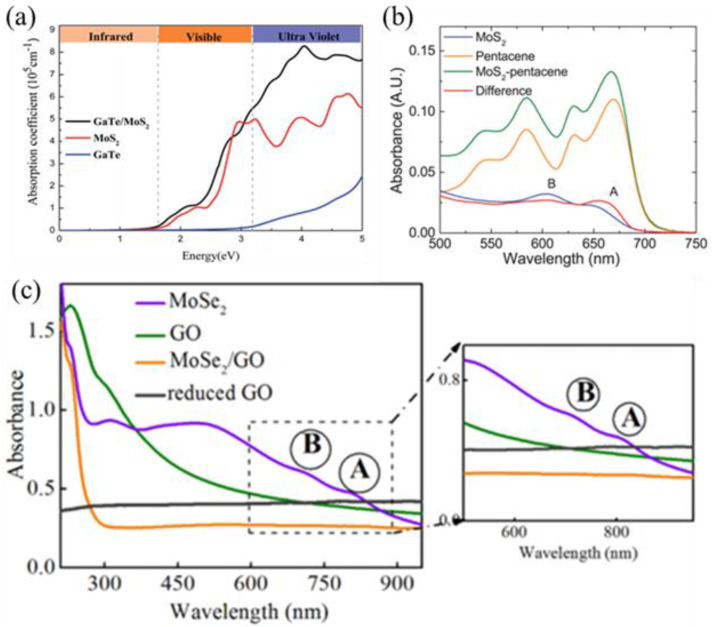
(**a**) Adsorption coefficients of the GaTe monolayer, MoS_2_ monolayer, and GaTe/MoS_2_ vdWH. Reprinted with permission from Ref. [49]. (**b**) Absorption spectra of MoS_2_-pentacene heterojunction. Reprinted with permission from Ref. [36]. Copyright 2017, American Chemical Society. (**c**) Optical absorption spectra of GO, MoSe_2_, and MoSe_2_/GO. Reprinted with permission from Ref. [85]. Copyright 2016, APS.

Absorption spectroscopy is another effective way to characterize light–matter interaction for revealing interfacial electronic coupling of vdWHs. The absorption spectrum is the spectrum of a substance that absorbs a photon and jumps from a lower energy level to a higher energy level. The absorption intensity of vdWHs is often the superposition of the absorption intensity of two materials. Guo et al. [48] found that the MoSSe monolayer has higher light absorption in the visible region, and the InSe monolayer has higher light absorption in the UV region. After the formation of vdWH, interlayer coupling occurs between the two monomolecular layers, and the reduction in the forbidden band gap produces charge transfer and enhanced light absorption. Therefore, MoSSe/InSe vdWHs have significantly improved light absorption, especially in the UV region. Similarly, as shown in Figure 5a, the optical absorption properties of GaTe/MoS_2_ vdWH in the visible and UV regions are significantly enhanced compared to the GaTe monolayer and MoS_2_ monolayer, indicating that the coupling between the monolayers leads to the band gap reduction and charge transfer, and thus the enhancement of the light absorption [49]. It has also been noted that the absorption spectrum of a vdWH is not only a superposition of the absorption spectra of two individual layers but sometimes reflects the interfacial coupling by the appearance of new absorption peaks or the peak shift of some original peaks due to the interlayer interactions. Therefore, it can also be used to characterize the coupling at the interface of vdWHs. For example, in the 2D MoS_2_/PbS heterojunction, PbS enhanced the light absorption of MoS_2_ and induced the optical peaks red shift slightly, which is probably derived from the quantum confinement of the charge carrier wave functions [83]. The red shift of the absorption peak is also believed to be evidence of the interfacial charge transfers by forming strongly coupled heterostructures [84]. As shown in Figure 5b, the A and B exciton peaks of monolayer MoS_2_ moved to a lower energy region after pentacene deposition, which is likely due to the formation of a depletion region in MoS_2_ [36]. In addition, the absorption spectrum also shows the appearance of new absorption peaks or the suppression of the original peaks, etc. Sharma et al. investigated the interlayer interaction between MoSe_2_ and GO by using absorption spectroscopy [85]. They found that the A and B exciton bands of MoSe_2_ are suppressed (as shown in the inset of Figure 5c). The combination of absorption and Raman spectroscopies can also be utilized to study the interfacial coupling in organic–inorganic vdWHs. After depositing various metal-phthalocyanine (MPc) on top of MoS_2_ to form a heterostructure, Amsterdam et al. systematically investigated the charge transfer in such vdWHs by using both Raman scattering and optical absorption spectroscopy [50]. It has been found that the interfacial charge transfer is sensitive to metal in the MPc molecules. Compared to Raman and PL spectroscopies, absorption spectroscopy indicates the first process of light–matter interaction and often provides a more direct description of light–matter interaction. Especially when used together with Raman and PL spectroscopies, it is capable of providing a comprehensive physical picture of interfacial coupling in vdWHs.

In summary, we have overviewed the applications of several optical microscopy-based steady-state characterization techniques such as Raman, PL, and absorption spectroscopy in the investigation of interfacial coupling in vdWHs. In practical applications, they were sometimes used together to study the intrinsic optical properties of vdWHs to provide a comprehensive picture of the light–matter interaction process. Just as discussed above, all these techniques are limited by the lateral resolution caused by the diffraction limit of light. Therefore, how to achieve higher resolution and maintain fast and efficient tests will be the main challenges for these OM-based techniques to provide more information about the interfacial coupling of vdWHs in the future [86].

### 2.2. Transient Optical Characterization

The above-mentioned characterization of steady-state optics can provide information such as many-body effects, interlayer exciton formation, and local stresses, but it is difficult to directly obtain the kinetics of exciton lifetimes and other dynamics of interlayer processes such as exciton diffusion length, mobility, and optical path changes under conditions of external field excitation. In this case, transient measurements are needed. Optoelectronic processes, such as charge transfer and separation, energy transfer, and carrier lifetime at the interface of vdWHs generally occur at the picosecond/nanosecond scale. Time-resolved photoluminescence spectroscopy (trPL) and transient absorption spectroscopy (TA) are two of the most common optical techniques to study these processes at present [18]. In addition, this paragraph also briefly introduces the time-resolved ESFG for probing time-dependent molecular conformation and interlayer electronic structure at the interfaces of vdWHs. Time-resolved photoluminescence spectroscopy is capable of providing the fluorescence lifetime by exciting the material with a short pulse of light and then recording the decay of fluorescence intensity with time. For type-II vdWHs, a staggered band alignment automatically occurs at the interface, leading to rapid charge separation of optically generated electron–hole pairs, formation of interlayer excitons, and stable trions, even at room temperature. In order to apply such vdWHs in future electronic and optoelectronics, it is necessary to achieve an overall understanding of the ultrafast carrier recovery time and the dynamics of these special excitons (interlayer excitons, biexcitons, and trions, etc.) in such a system.

**Figure 6:**
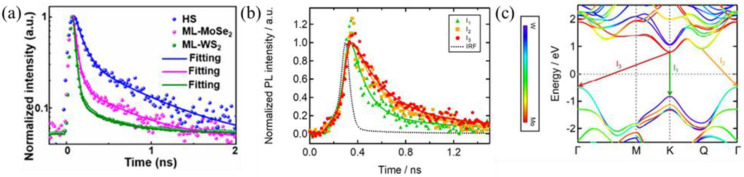
(**a**) Time-resolved PL decay curves for MoSe_2_ monolayer, WS_2_ monolayer, and WS_2_/MoSe_2_ Hs. Reprinted with permission from Ref. [40]. Copyright 2021, American Chemical Society. (**b**) Time-resolved PL intensity of hBN/WS_2_/MoS_2_/hBN [87]. (**c**) DFT band structure of WS_2_/MoS_2_ Hs [87]. Reprinted with permission from Ref. [87]. Copyright 2018, American Chemical Society.

The trPL spectroscopy can be used to measure carrier lifetime and confirm the formation of interlayer excitons. As shown in Figure 6a, we obtained a faster decay lifetime of 32 and 44 ps for WS_2_ and MoSe_2_, respectively, and a slower lifetime of 93 ps for the WS_2_/MoSe_2_ heterostructure derived from interlayer exciton in our previous work [40]. Okada et al. also revealed that the relaxation times of the indirect interlayer excitons are longer than direct interlayer excitons by room-temperature trPL [87], as shown in Figure 6b,c.

**Figure 7:**
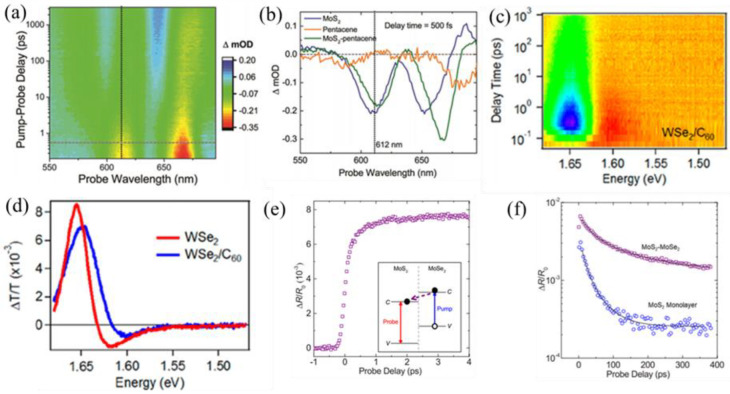
(**a**) Two-dimensional transient absorption spectra for the MoS_2_-pentacene heterojunction [36]. (**b**) Transient absorption spectroscopy of the MoS_2_-pentacene heterojunction. Reprinted with permission from Ref. [36]. Copyright 2017, American Chemical Society. (**c**) Two-dimensional color plot of TA spectra of the WSe_2_/C_60_ heterojunction [54]. (**d**) TA spectra of the WSe_2_ monolayer and WSe_2_/C_60_ heterojunction. Reprinted with permission from Ref. [54]. Copyright 2021, American Chemical Society. (**e**) The electron density after excitation of photocarriers in MoS_2_. Built-in illustration showing pump–probe configuration. [91]. **(f**) The electron density of heterostructures and MoS_2_ monolayer. Adapted with permission from Ref. [91]. Copyright 2014, American Chemical Society.

Transient absorption spectroscopy is also called the pump–probe technique. It is a more direct and time-resolved method of studying the photocarrier dynamics in heterostructures [88,89]. In ultrafast pump–probe experiments, two ultrashort laser pulses of different intensities and wavelengths are used, with the strong light as the pump light and the weak light as the probe light, and then both beams are focused on the same location on the sample. In the previous study, Hong et al. prepared MoS_2_/WS_2_ van der Waals heterostructures. By combining photoluminescence mapping with transient absorption measurements, it was determined that holes could be transferred from the photoexcited MoS_2_ layer to the WS_2_ layer within 50 fs [51]. In the heterostructure formed by MoS_2_ and ReS_2_, He et al. found that the transfer time of photocarriers was about 1 ps [52]. Peng et al. determined that in a 2D MoS_2_/WSe_2_ heterostructure, photocarriers injected into WSe_2_ are transferred to MoS_2_ within 470 fs, and the photo-generated excitons effectively dissociate into free electrons and holes and are transferred to different layers [53]. Ultrafast charge transfer has been reported at the interfaces of organic–inorganic van der Waals heterostructures. For instance, Liu et al. protonated 2DPI-graphene (2DPI-G) vdWHs and found that the generated H-2DPI- G can exhibit significant ultra-high speed charge transfer within 60 fs guided by interlayer cation-π interaction. This result can be comparable to the fastest charge transfer reported in inorganic 2D vdWHs [90]. Transient absorption spectra can also be detected by using ultrafast white light as probe light while using monochromatic laser excitation. Homan et al. used 535 nm light as the pump wavelength and probed the sample with continuous white light (Figure 7a,b) [36]. The dissociated MoS_2_ exciton was found to be transferred to the pentacene in the pentacene-MoS_2_ heterostructure at a 6.7 ps time scale. The charge-separated state survives for 5.1 ns. The results show that the hole transfer rate from molybdenum disulfide to pentacene is 50%, and the remaining holes are trapped due to surface defects. This offers important prospects for the applications of such hybrid vdWHs in photovoltaics, photodetectors, and related optoelectronic techniques. Zhao et al. studied the carrier dynamics in WSe_2_/C_60_ heterostructures by using 725 nm light as the pump light and continuous white light as the probe light [54]. The relevant TA spectra are shown in Figure 7c,d. By using ultrafast spectroscopy, the exciton in WSe_2_ was found to be dissociated by ultrafast (~1 ps) electron transfer to C_60_ and in a nanosecond-long charge separation process. Due to the suppressed electron–hole exchange interaction after exciton dissociation, the hole in WSe_2_ has a spin/valley polarization lifetime of 60 ps at room temperature, which is two orders of magnitude longer than that of the hole in an isolated WSe_2_ monolayer. In addition to probing the length of carrier lifetime, the TA method can be used to purposefully excite and probe special electronic states, such as special excitons. Heterostructures formed by MLs of MoS_2_ and MoSe_2_ were studied by Ceballos et al. [91]. The formation of interlayer excitons was demonstrated by the choice of excitation and detection light, and the lifetime of interlayer excitons was fitted. As shown in Figure 7e, by probing the MoS_2_ resonance and monitoring the electron density in MoS_2_, it can be concluded that the charge transfer process occurs on the sub-picosecond time scale. Moreover, measurements performed on longer time scales show (Figure 7f) that indirect excitons are formed due to the separation of electrons and holes in the two layers with longer lifetimes than in MoS_2_ MLs. In addition, the time-resolved pump–probe measurements have been developed. It can be used to extract the spatial diffusion length, carrier mobility, and other semiconductor-related parameters of photoexcited carriers in vdWHs. Liu et al. [92] used this technique to study the charge transfer characteristics of two heterostructures, WS_2_/Bi_2_O_2_Se and MoS_2_/Bi_2_O_2_Se. Time-resolved pump–probe measurements showed interlayer charge transfer with near unit transfer efficiency and formation of interlayer excitons with long, complex lifetime in these two heterostructures.

Transient absorption spectroscopy is capable of providing an ultrafast laser pump–probe detection for studying carrier dynamics such as relaxation processes in excited states and interfacial charge transfer as well as optical pathways in vdWHs. Most of the fast lasers used now are in the levels of hundreds of femtoseconds to picoseconds. However, a very important process related to the interfacial coupling in vdWHs, phonon relaxation, usually decays very fast and can hardly be captured by current measurement conditions. Therefore, the development of a faster laser will strongly promote the research progress and broaden the understanding of vdW interaction in such Hs.

In addition to the trPL and TA methods described above, second-harmonic generation (SHG) is a highly sensitive fine magnetic order probe that opens up the possibility of two-dimensional magnets for applications in nonlinear and nonreciprocal optics. Nonreciprocal second-order nonlinear optical effects appearing in bilayer CrI_3_ were reported by Sun et al. [93]. It is shown that although the parent lattice of bilayer CrI_3_ is centrosymmetric and does not contribute to the SHG signal, the observed giant nonreciprocal SHG originates from laminar antiferromagnetic ordering only, breaking both spatial inversion symmetry and temporal inversion symmetry. Yao et al. used ultrashort laser pulses to excite electrons and holes in the heterogeneous structure of MoS_2_/WS_2_ [55]. The electrons and holes are separated from the two monomolecular layers, generating an electric field that causes the incident fundamental wave to produce a second harmonic. The space charge field generated by the charge transfer decays with a time constant of 125 ps. The long-time constant is consistent with the space charge field lifetime, which is obtained from time-varying differential SHG measurements. This work provides an all-optical method for studying charge transfer with high temporal resolution. In addition, a new set of methods, i.e., sum frequency generation (SFG), electronic sum-frequency generation (ESFG) and time-resolved ESFG (TR ESFG) spectroscopies have been developed to provide interfacial molecular conformation, electronic structure and the dynamics of 2D materials [94,95,96]. ESFG utilizes narrow-band and broadband visible (including NIR) femtosecond pulses to provide high-quality interface-selective electron spectroscopy. TR-ESFG combines femtosecond optical excitation and ESFG measurements to provide interface-selective time-resolved electron spectroscopy to study the ultrafast dynamics of interfacial molecules [97,98,99,100]. Li et al. demonstrated that electronic sum-frequency generation (ESFG) spectroscopy could be used to determine the electronic structure of interfacial molecules at a solid/solid interface [99]. It is studied by ESFG that the conformation of P3HT at the interface of P3HT/n-doped Si (111) is more homogeneous than the native conformation. There are still rare reports of applying such techniques on both inorganic 2D/2D heterostructures and inorganic–organic vdWHs. We believe as by combining such techniques together with trPL and TA, etc., techniques, more insights about the relationship between interfacial coupling and device performance of vdWHs will be revealed.

In summary, transient spectroscopy has been rapidly developed in recent years. The development of ultrafast spectroscopy techniques has also accelerated the combination with other techniques. Some of the more common combined techniques are electron diffraction, AFM, microwave techniques, angle-resolved photoemission spectroscopy (ARPES), STM, and other techniques. The applications of these techniques will provide more insights into the interfacial coupling in vdWHs. In addition, in practical applications, in situ transient absorption spectroscopic tests and other steady-state spectroscopic experiments of vdWHs-based devices under performance will allow a better understanding of the microscopic mechanisms for nanodevices and aid in the optimization of device structures in the future.

### 2.3. Other Characterizations

In addition to the several optical characterization methods described above, there are some other characterization techniques, such as multi-function SPM (including KPFM, AFM, and STM), ARPES, and transmission electron microscopy (TEM), etc., have proven to be very powerful tools to study the interfacial coupling of vdWHs. Although optical methods discussed above are capable of providing a lot of useful information, especially ultrafast time resolution measurements, about the interfacial coupling of vdWHs, they are still limited in their spatial resolution since the beam size of the laser can usually be focused on a few hundred nanometers to microns. Compared to the optical methods, SPM techniques, ARPES, and TEM have a much higher spatial resolution, but a high vacuum is usually required.

KPFM can be used to provide the surface potential, local states, and working function of vdWHs. For instance, KPFM has been utilized by Huang et al. to prove that separating photogenerated holes toward ZnPc and away from the trap states in MoS_2_ in MoS_2_/ZnPc vdWH can effectively suppress unsolved persistent photo-conductance in as-built photodetectors [23]. Except for KPFM, electrostatic force microscopy (EFM) is also very useful for investigating the local electrical information at the surface of vdWHs, including charge distribution, carrier density, and so on [101]. For example, EFM measurements have been carried out by Jariwala et al. to confirm directly the existence of band bending in the pentacene/MoS_2_ junction region, which aids in carrier separation in such vdWHs-based photovoltaics [102]. It has been noted that in the cases above, both KPFM and EFM operate under an ambient atmosphere; therefore, their spatial resolutions are largely limited by the existence of adsorbed molecules, contamination, and water films at the surface; even probes with ultrasharp tips were used during the measurements. KPFM and EFM under high vacuum enable better spatial resolution; however, the surface of the samples should be super clean, and annealing at high temperature is usually needed to achieve high vacuum conditions in the chamber. STM is also a powerful tool that can be utilized to investigate the interfacial coupling of vdWHs. Especially, ultrahigh-vacuum STM, as equipped with scanning tunneling spectroscopy (STS), can achieve the measurement of the electronic structure of vdWHs with a spatial resolution even down to the atomic level. For example, Ospina et al. found through theoretical STM images that the electronic structure of hBN/BP was found to change depending on the type of defects introduced. These defects can be identified in STM experiments even if they are located below the phosphorus layer [103]. Chiu et al. used microbeam X-ray photoelectron spectroscopy and scanning tunneling microscopy/spectroscopy to determine the energy band offsets in the transition metal dihalide heterostructures. They determined the type-Ⅱ alignment between MoS_2_ and WSe_2_ with values of 0.83 eV and 0.76 eV for the valence band and conduction band offsets, respectively. The valence band offsets obtained from density flooding theory are in agreement with the experimental results [104]. Furthermore, combining STM and STS measurements, Parks et al. discovered that the defects in the MoS_2_ can be effectively passivated by depositing TiOPc molecules on top of MoS_2_ [105].

In addition, ARPES is the only experimental technique that can accurately characterize band structures in momentum space directly. This technique can distinguish clear band structures, although a high vacuum is usually required during the ARPES measurement. Aeschlimann et al. found that the lifetime of the charge separation transient in the WS_2_/graphene heterostructure was close to 1 ps. They attributed this finding to spatial differences in scattered phases caused by the relative alignment of WS_2_ and graphene bands revealed by high-resolution ARPES [56].

Moreover, TEM is a very powerful tool to characterize interfacial coupling in vdWHs with high spatial resolution, especially as equipped with energy spectroscopy, such as electron energy dissipation spectroscopy (EDS) and electron energy loss spectroscopy (EELS). For example, Kafle et al. determined that MoS_2_ is a single crystal while ZnPc is a polycrystal in MoS_2_-ZnPc heterostructures [57]. Wang et al. observed that the diameter of the WS_2_/MoS_2_ vdWHs nanoscroll is about 100 nm by TEM. The central region is darker than the edge of the nanoscroll, indicating that WS_2_/MoS_2_ vdWHs nanoscroll has a dense scrolled structure [58]. The VSe_2_/WSe_2_ vdWHs were studied by Li et al. [106]. Detailed atomic structure characterization of VSe_2_/WSe_2_ vdWHs was performed by a combination of TEM, scanning transmission electron(STEM) microscopy, and EDS. In addition, X-ray diffraction (XRD), X-ray photoelectron spectroscopy (XPS), and four-wave mixing (FWM) spectroscopy et al. have been applied in the characterization of van der Waals heterostructures [107] to provide overall information about the interfacial coupling.

The above techniques have been commonly used to characterize the interfacial coupling of vdWHs. In most practical cases, it is hard to obtain all the information by only one technique; therefore, the combination of multiple techniques is very necessary to obtain more comprehensive information about the interfacial coupling in vdWHs. More importantly, except the development of advanced characterization techniques, new calculations methods, as well as the development of the new theory in this field, will be in urgent demand to either provide more accurate calculation results to support the experimental data or to predict the new physics in this fields in the future.

## 3. Modulation of Interfacial Coupling

As stated by Nobel Prize winner Herbert Kroemer, “the interface is the device” [108]. Indeed, interfaces are ubiquitous in electronic and optoelectronic devices and dominate device performance. Therefore, it is essential to gain a basic understanding of the interface, develop rational modulation methods to tune the interface effectively, and thus optimize the device structure and control the performance of devices eventually. In particular, interfacial coupling in vdWHs dominates the interfacial properties and holds a particular interest in optoelectronics. Previously, several mechanisms, such as the delocalization of electron wavefunction in momentum space [109], coherence enhancement [110], Coulomb potential enhancement [111], resonance transfer to higher energy states [109], phonon assistance [112], new mechanisms for hot electron/hot hole relaxation [113], etc., have been proposed to explain the fascinating phenomena observed in vdWHs. Meanwhile, many research groups reported that the interfacial electronic structures, such as bandgap, exciton formation, spin, etc., could be controlled experimentally by materials selection, as well as by applying external fields. Therefore, electron–hole behaviors across the vdWHs could be well tailored, either to generate photons with a designed frequency or to separate to form free carriers for photodetection or photovoltaics. Except for the material selection, various modulation methods, including interfacial strain engineering, structural optimization, and applied external fields, have been developed in the last decade to make the rational design of vdWHs-based nanodevices possible [40,114]. Herein, we overview the timely research progress in the effective approaches to modulate the interfacial coupling in vdWHs for future nanodevices. The modulation methods by applied fields, such as electric, optical, and mechanical fields and stacking angle [115], will be briefly reviewed in this section. According to these interfacial engineering methods, the interfacial properties, including carrier generation, separation, transport, and charge transfer pathways and dynamics, can be efficiently tuned.

### 3.1. Strain Engineering Modulation

**Figure 8:**
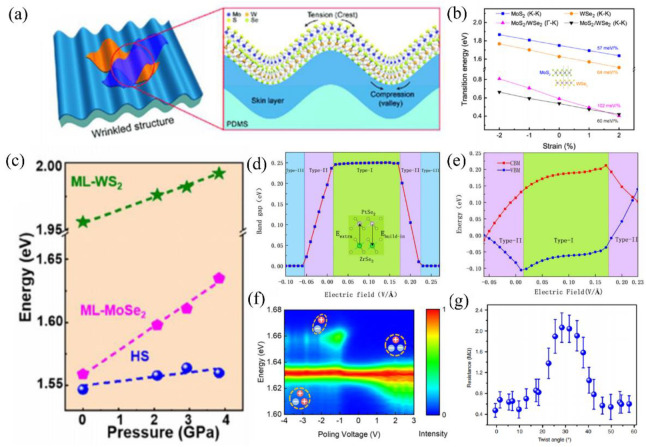
(**a**) Schematic diagram of the folded MoS_2_/WSe_2_ heterostructure [117]. (**b**) Monolayer MoS_2_ and monolayer WSe_2_, MoS_2_/WSe_2_ electron leap energy as a function of strain. The stacking order of AB layers is shown in the inset. Reprinted with permission from Ref. [117]. Copyright 2021, American Chemical Society. (**c**) Variation of peak energy of A_MoSe2_ and A_WS2_ with pressure. Reprinted with permission from Ref. [40]. Copyright 2021, American Chemical Society. (**d**) Band gap variation of PtSe_2_/ZrSe_2_ vdWHs under different electric fields [61]. (**e**) Band edges variation of PtSe_2_/ZrSe_2_ vdWHs under different electric fields. Reprinted with permission from Ref. [61]. Copyright 2020, Elsevier. (**f**) PL spectrum of MoSe_2_ at polarization voltages from −4V to +3V. Reprinted with permission from Ref. [124]. Copyright 2021, American Chemical Society. (**g**) Resistances of MoS_2_/Gr heterostructures with different twisted angles [125].

Efficient interfacial charge transport is essential for the construction of high-performance optoelectronic devices based on vdWHs. Due to the excellent mechanical properties of 2D materials, strain engineering is considered an effective means to modulate the energy band structure of 2D materials and thus the interfacial transport of vdWHs [116]. A lot of 2D materials have high in-plane mechanical strength and very small bending stiffness, which makes them particularly suitable for strain engineering [117]. For example, monolayer MoS_2_ will undergo a direct to indirect band gap transition at 2% tensile uniaxial strain and a semiconductor–metal transition at 10–15% tensile biaxial strain [118,119]. Cho et al. [117] also investigated the strain tunability of the electronic structure in the MoS_2_/WSe_2_ heterostructure (the sample schematic is shown in Figure 8a) by placing the heterostructure in a wrinkled PDMS substrate. Effective strain tuning of the Γ-K interlayer excitons can be observed by applying ~107 meV/% uniaxial strain. They also found that momentum–space indirect Γ-K interlayer excitons are approximately twice that of the constituent intralayer excitons (As shown in Figure 8b). According to their results, the interlayer coupling of such vdWHs can be directly tuned in the local strain structure. Except for the experimental reports, many calculations have also been performed either to support the experimental results or to predict the modulation of strain on interfacial coupling in vdWHs too. For example, the density functional theory (DFT) calculations that have been carried out in this work to investigate the experimental observation are consistent with the calculations [120]. In addition, Guo et al. systematically evaluated the modulation of the electronic structure, optical properties, and mechanical properties of MXene/Blue P vdWHs under strain by density generalized theory [59]. It is predicted that the type-І heterostructure of Zr_2_CO_2_/Blue P vdWHs can be transformed into the type-Ⅱ heterostructure under appropriate strain conditions, which is favorable for charge separation. These experimental and theoretical works highlight the potential of strain engineering in the interfacial coupling of vdWHs. Except for applying a tensile strain, applying high pressure directly on the vdWHs has also been carried out as an effective method to modulate the interfacial coupling in vdWHs [40,121]. In our previous work, we reported an effective modulation of interfacial coupling in high-quality WS_2_/MoSe_2_ heterostructure by applying hydrostatic pressure through diamond anvil grooves. It is interesting that the transition from intralayer excitons to interlayer excitons can be efficiently tuned under high pressure [40]. Especially, the energy of interlayer exciton in this vdWH is very stable and almost kept unchanged under high pressure (Figure 8c). Theoretical calculations also reveal that the enhanced interlayer interaction in WS_2_/MoSe_2_ heterostructure under pressure leads to enhanced interlayer exciton behavior. This work provides an effective strategy to study the interlayer interactions of vdWHs by pressure modulation. Therefore, no matter whether applying tensile strain or high pressure or placing the vdWHs on a patterned substrate, the interfacial coupling, including interfacial electronic structure, exciton formation, and even moiré potential landscape [122], can be effectively modulated. All of these results establish that strain engineering is an effective tool to tailor interfacial coupling and explore new physics of vdWHs for optoelectronics on demand [123].

The above research shows that after applying stress to the sample, by changing the magnitude and direction of the stress, the band gaps will change accordingly. The vdWHs properties will also be effectively tuned. Up to now, there have been few studies on the effect of strain on interlayer coupling in vdWHs. Especially, moiré potential was revealed as local lattice distortion; therefore, understanding and introducing the strain field engineering to achieve a similar effect of strong moiré potential in the interfacial coupling of vdWHs will provide guidance for the design of optoelectronic devices with an optimized performance by local stain engineering.

### 3.2. Electric Field Modulation

Except for strain engineering, the modulation of the interfacial coupling of vdWHs by applying an electric field has been reported as well. As early as 2009, bilayer graphene showed a significant band gap under a large electric field [126]. Similarly, the band structures of vdWHs can also be effectively adjusted under an electric field. Generally, the realization of the electronic control of interfacial coupling in vdWHs required the nanoscale control of charge doping in these materials. Theoretical predictions have been carried out by many research groups to examine the tunability of interfacial coupling in vdWHs by adding electric bias. For example, Wei et al. calculated the band gap of the phosphorene/PbI_2_ heterojunction, which varied from 0 to 0.90/1.54 eV in density functional theory with applied electric field [60]. In addition, some research groups examined the electric effect on vdWHs by using STM or conductive AFM tips or SEM electron beams [114,127]. These methods benefit from having no contaminations from the complicated device fabrication processes; thus, they are perfect for the experimental exploration of electrical control in vdWHs. For instance, the Crommie group achieved a nanoscale visualization of the electrical doping of graphene/boron nitride vdWHs by using an STM tip [127]. Some attempts have also been made to obtain effective nanoscale doping in 2D materials by using focused electron-beam irradiation in SEM [114,128]. In 2020, the Zettl group reported a fully reversible high-resolution electron beam doping on graphene/MoS_2_ vdWHs. By employing a BN-encapsulated geometry, direct electron-beam irradiation could be effectively avoided, and the doped vdWHs demonstrated a previously inaccessible regime of high carrier concentration and high mobility, even at room temperature [114]. Although these techniques offer an efficient route for tailoring the doping in vdWHs at the nanoscale, they are not compatible with large-scale doping operations. In order to make the approach technologically practical, the electrical-modulation methods need to be applied in higher levels of functional systems, such as logic circuits. Therefore, an FET-based device structure has been designed to evaluate the effect of electric bias on vdWHs. For instance, one can see that the relationship between the band gap/band edges of PtSe_2_/ZrSe_2_ vdW heterojunction with an external electric field in Figure 8d,e [61], which directly enhanced the versatile applications of PtSe_2_/ZrSe_2_ vdWHs in electronics and optoelectronics under an external electric field. Among these research works, FET-based device structures were usually fabricated to apply an external electric field on the vdWHs through the top gate or back gate using normal dielectrics. However, for 2D materials, it is challenging to achieve a high doping level by using normal gate dielectrics due to their atomically thin structure and limited space for the dopants with sufficient numbers without causing huge damage to the structure. Therefore, electrostatic gating, e.g., ionic liquid and some ferroelectric substrates, have also been utilized to alter the properties of vdWHs by tuning the sign and concentration of electrical doping to a high doping level without changing the chemical compositions of the vdWHs [129,130]. Especially, along with the recent discovery of 2D ferroelectric materials, similar van der Waals structures can be built on top of 2D ferroelectric substrates [130]. Usually, the spontaneous electrical polarization of ferroelectric materials can be reversed by applying an intensified electric field; thus, it can be performed locally to create a spatial domain pattern of positive or negative polar surfaces and provides a strategy for lateral modulation in TMD to generate an electrostatic driven p-n homojunction. For example, Mao et al. found that the electric polarization of CIPS leads to continuous, large electron modulation in the monolayer MoSe_2_ in the MoSe_2_/CuInP_2_S_6_ (CIPS) 2D ferroelectric heterostructure. Using saturated ferroelectric polarization of CIPS, electron-doped or hole-doped MoSe_2_ can be achieved in a single device (Figure 8f). These devices have non-volatile behavior for up to 3 months. The results provide a new pathway for low-power and long-time tunable optoelectronic devices [124]. Wen et al. also found that MoSe_2_ and WSe_2_ can bind to the ferroelectric lithium niobate surface and that the domain orientation of the LiNbO_3_ has a strong effect on the TMD optoelectronic properties. Monolayer TMD sheets can be designed with underlying ferroelectric domains to create p-n homogeneous junctions that are sensitive to laser power and ambient temperature. This approach lays the foundation for creating active, electrically-driven, and controllable optoelectronic elements on a LiNbO_3_-integrated photonic platform [131]. In summary, the external electric field can modulate the height of the Schottky barrier, the type of interfacial contact, and the number and direction of the interfacial charge transfer. It can also realize the reversible flip of the electric field at the heterostructures interface so that its optical response is continuously adjustable. Electric field modulation has thus become one of the current hot spots of research. However, up to now, there are still few reports regarding the electrostatic field regulation on organic–inorganic heterojunctions based on 2D materials and organic small molecules, except organic–inorganic hybrid perovskite [132]. Moreover, such electric field modulation is often limited by dielectric breakdown; other modulation methods are also needed to controllably tune the interfacial coupling in vdWHs.

### 3.3. Optical Field Modulation

Optical modulators are increasingly in demand for existing and emerging technologies, and high-performance optical modulation has become a hot topic of current research. It is significant to develop high-performance optical modulation with compact, economical, efficient, fast, and broadband optical interconnects [133]. Except for the electrical modulation, the photo-induced tuning of vdWHs was also explored in recent years [134,135]. Similar to electrical doping using voltage pulses from the STM tip [136], photo-induced doping can also be achieved through different optical excitation. The F. Wang group reported that the modulation doping of graphene could be effectively controlled through the optical excitation of BN in graphene/BN heterostructures. It is interesting to see that this photo-doping in graphene/BN is more than 1000 times stronger than that in undoped graphene/BN devices [134]. Except for the analogous modulation doping effect, strong light–matter interaction in 2D materials also triggers a lot of fascinating physical phenomena. For instance, 2D materials hold great potential to provide extremely high optical nonlinearity [137] for manipulating ultrafast light at deep subwavelengths or at the nanoscale [138], thus providing new opportunities for smaller and faster optical modulators and related devices. Optical modulation to control the strength, phase, or polarization of 2D materials has aroused more and more attention in recent years [133,139]. For example, laser pulse excitation can significantly change the carrier density, thus changing the complex refractive index of the material so as to adjust the optical response of the material to obtain better performance. Han et al. demonstrated a graphene/C_60_/pentacene vertical heterostructure [62]. In the infrared light modulation of graphene/thin-layer C_60_ (5 nm)/pentacene, the photoresponsivity was increased from 3425 A/W to 7673 A/W. Furthermore, visible light modulation provides a tunable bandwidth from 10 to 3 × 10^3^ Hz for tuning the infrared photocurrent. The introduction of vdWHs greatly enhances spectral response range and optical response, while the long lifetime of interlayer excitons and the stability at room temperature make it possible to manipulate excitons through external electric and optical fields, which opens the way for the development of new exciton devices. However, studies using light modulation to tune the interlayer interaction between two stacked 2D layers to create new material systems with strongly enhanced light–matter interactions have not been reported as far as we know. It is worth noting that for bilayer structures consisting of 2D materials with large spin–orbit coupling strengths or large inverse Rashba–Edelstein coefficients and magnetic materials, spin–charge conversion can be achieved by microwave excitation of the magnetron on the magnetic substrate, such as graphene-YIG [140] and MoS_2_-YIG [141].

### 3.4. “Twisted Angle” Modulation

Except for the modulation by applying external fields discussed above, the properties of vdWHs can also be tuned by stacking methods. When two different monolayer materials are contacted to form a bilayer, electrons are trapped by spatially periodic “moiré potential” and can no longer move freely along the atomic plane. At the very beginning, moiré potentials have been predicted to modify the physical properties of such a bilayer. Inspired by the discovery of “magic angle” in bilayer graphene [142,143], the interlayer coupling of various van der Waals heterojunctions was also found to be effectively tuned by adjusting the twisted angle of the two monolayers of the materials since moiré potential can be different as the layers have different orientations. “Magic angle” can achieve superb electrical properties without changing the material, only by changing the external force. Therefore, tremendous reports have been published about the exploration of new physics such as unconventional superconductivity, moiré excitons, tunneling conductivity, nonlinear optics, structural super lubrication, and so on, in 2D systems by tuning the twisted angle in the past few years [144,145,146,147,148]. It has been noted that there are many review papers discussing the “magic angle” in bilayer graphene [142,149]; therefore, we will only focus on the modulation of the interfacial coupling in van der Waals heterostructures formed by TMD materials and graphene by twisted angle.

Similar to the “magic angle” effect in bilayer graphene, Liao et al. found that the vertical electric conductivity behavior of MoS_2_/graphene heterostructures strongly depends on the stacking angle of the heterostructure, and its vertical conductivity increases monotonically with the stacking angle from 0° to 30°. The vertical conductivity of a 30° stacking angle heterostructure is approximately five times higher than that of a 0° stacking angle. (As shown in Figure 8g) Density functional theory (DFT) simulations suggest that such variation in conductivity arises from the difference in transmission coefficients of heterostructures with different twist angles [125]. In addition, studies have also shown great opportunities for modulation of the optical properties of vdWHs by changing the twisted angle of two monolayer materials. Cao et al. discovered that when two parallel graphene layers are stacked at a delicate angle of about 1.1°, a superconducting effect occurs. It shows that twisted bilayer graphene is an ideal material for studying strongly correlated phenomena [142,143]. They subsequently discovered moiré superconductors in magic-angle twisted trilayer graphene, and the tunability of this system in terms of electronic structure and superconductivity properties is superior to that of magic-angle twisted bilayer graphene [150]. They further investigated the broken symmetry many-body ground state of magic-angle twisted bilayer graphene and its nontrivial topology using thermodynamic and transport measurements. The importance of favoring Hund’s coupling in the many-body ground state is demonstrated [151]. Seyler et al. report experimental evidence of interlayer valley excitons captured in moiré potentials in MoSe_2_/WSe_2_ heterostructure. This work provides an opportunity to control the two-dimensional moiré optics by varying the twist angle [63]. Similarly, Alexeev et al. demonstrated through the heterostructure of MoSe_2_/WS_2_ that exciton energy bands can hybridize, leading to resonant enhancement of the moiré superlattice effect. For heterostructures with single-molecule layer pairs in close alignment, resonant mixing of electronic states leads to a significant effect of geometrically patterned moiré of the heterostructure on the dispersion and spectrum of hybridized excitons [147].

However, there are many problems with the means of “twisted angle” modulation. For example, the unevenness of two-dimensional materials at the atomic level can lead to multiple interfacial unevenness after stacking of bilayers or more, and the effect of this situation is amplified in heterostructures. Second, it is difficult to achieve precise control of the angle when modulating the angle of bilayer or multilayer 2D materials, while the effect on the performance of the heterostructures is unknown. Finally, the control of small angles is difficult, and how to control the angle of the torsion angle in a stable and simple way is a problem that needs to be overcome in the near future.

In conclusion, the charge and energy transfer in vdWHs is mainly determined by the energy band alignment and is also greatly influenced by the dielectric function, stacking method, crystal orientation, and external fields such as electric/optical/magnetic. Therefore, the modulation of interfacial coupling by using strain engineering, electric field, optical field, as well as changing stacking angle will provide various methods for applications of vdWHs in ultrafast and high-performance optoelectronic devices eventually.

## 4. Conclusions

In summary, this paper discusses the characterization and modulation methods of interfacial coupling at vdWHs. For steady-state characterization, it is generally believed that if the vdWHs have strong interlayer coupling, the Raman, absorption, and PL peaks will shift, even appearing as new peaks, and the PL strength will be quenched due to the emergence of the interlayer exciton. Further by TRPL, TA, Pump–probe spectroscopy, etc., the transient characterization can obtain the ultrafast dynamics of the interfacial molecules directly, such as the kinetics of exciton lifetimes, exciton diffusion length, carrier mobility, and so on. Of course, SHG, KPFM, TRESFG, ARPES, and other characterization methods also play an important role in verifying interlayer coupling. However, the in situ characterization of heterostructure-based devices is still rare; therefore, developing an accurate characterization technique to provide an in situ characterization of interfacial coupling in vdWH-based nanodevices under performance is essential and necessary. In addition, modulation strategies of interfacial coupling, including electrical, optical, mechanical fields, and the twisted angle between two layers, are very significant in optimizing the physical properties and thus improve the device performance based on vdWHs.

Except for the experimental work, a lot of calculation research was carried out simultaneously to predict or support the experimental results in this field. For instance, Xia et al. calculated the electronic band structure of the WSe_2_/MoSe_2_ heterostructures as a function of pressure by density functional theory with Perdew–Burke–Ernzerhof potential and obtained good agreement with the experiments [121]. Li et al. used first-principles calculations to predict that the MoS_2_/SnS heterostructure was a stable interface and that a small band gap (about 0.29 eV) could absorb the entire visible region [152]. Considering most of the heterojunctions mentioned in this paper are based on 2D inorganic-null models, we believe that the characterization and modulation of organic–inorganic heterojunctions are also worth exploring experimentally and theoretically. In addition, new phenomena generated by interlayer coupling need more theoretical calculations to predict. Moreover, it is also very important to integrate 2D materials and heterojunctions into industry for practical applications.

## Figures and Tables

**Table 1:** The types of vdWHs arrangement mentioned in the review.

Heterostructures	Band-Alignment Type	Reference
MoSe_2_/CuPc	type-II	[38]
hBN/WS_2_	type-I	[39]
WS_2_/MoSe_2_	type-II	[40]
MoS_2_/WSe_2_	type-II	[41]
MoS_2_/TaS_2_	type-II	[42]
WS_2_/ReSe_2_	type-II	[43]
2L-α-In_2_Se_3_/1L-MoSe_2_	type-I	[44]
Bulklike In_2_Se_3_/1L MoSe_2_	type-II	[44]
MoS_2_/pentacene	type-II	[36]
WS_2_/MoS_2_	type-II	[45]
MoS_2_/PbI_2_	type-I	[46]
WS_2_/PbI_2_	type-II	[46]
WSe_2_/PbI_2_	type-II	[46]
MoS_2_/GaS	type-II	[47]
S3-MoSSe/InSe	type-I	[48]
Se5-MoSSe/InSe	type-II	[48]
GaTe/MoS_2_	type-II	[49]
H_2_Pc/MoS_2_	type-II	[50]
MoS_2_/WS_2_	type-II	[51]
MoS_2_/ReS_2_	type-I	[52]
MoS_2_/WSe_2_	type-II	[53]
WSe_2_/C_60_	type-II	[54]
MoS_2_ and WSe_2_	type-II	[55]
WS_2_/graphene	type-II	[56]
MoS_2_-ZnPc	type-II	[57]
WS_2_/MoS_2_	type-II	[58]
MXene/Blue P	type-Ⅰ/type-II	[59]
phosphorene/PbI_2_	type-II	[60]
PtSe_2_/ZrSe_2_	type-Ⅰ/type-II	[61]
graphene/C_60_/pentacene	type-II	[62]
MoSe_2_/WSe_2_	type-II	[63]

## Data Availability

Not applicable.
